# Application of a simplified transesophageal echocardiography examination sequence in high-risk cardiac surgery

**DOI:** 10.1186/s13063-024-08338-9

**Published:** 2024-08-13

**Authors:** Chunrong Wang, Yuan Tian, Bing Bai, Kai He, Haisong Lu, Chunhua Yu, Qi Miao

**Affiliations:** 1 Department of Anesthesiology, Peking Union Medical College Hospital, Chinese Academy of Medical Sciences, Beijing, China; 2 Department of Cardiac Surgery, Peking Union Medical College Hospital, Chinese Academy of Medical Sciences, Beijing, China

**Keywords:** Cardiac surgery, Transesophageal echocardiography, Cardiopulmonary bypass, Hemodynamic instability

## Abstract

**Background:**

In cardiac surgical procedures, patients carrying high-risk profiles are prone to encompass complicated cardiopulmonary bypass (CPB) separation. Intraoperative transesophageal echocardiography (TEE), a readily available tool, is utilized to detect cardiac structural and functional pathologies as well as to facilitate clinical management of CPB separation, especially in the episodes of hemodynamic compromise. However, the conventional TEE examination, always performed in a liberal fashion without any restriction of view acquisition, is relatively time-consuming; there appear its flaws in the context of critically severe status. We therefore developed the perioperative rescue transesophageal echocardiography (PReTEE), a simplified three-view TEE protocol consisting of midesophageal four chamber, midesophageal left ventricular long axis, and transgastric short axis.

**Methods:**

This is a single-center and randomized controlled trial which will be implemented in Peking Union Medical College Hospital, Beijing, China. A total of 46 TEE scans are schemed to be performed by 6 operators participating in and randomly assigned to either the PReTEE or the conventional TEE group. This study is purposed to investigate whether the efficiency of discriminating leading causes of difficult CPB wean-off can be significantly improved via an abbreviated sequence of TEE views. The primary outcome of interest is the difference between the groups of PReTEE and the conventional TEE in the successful discrimination of etiologies in specified 120 s. Cox proportional hazards model will be further employed to calculate the outcome difference.

**Discussion:**

The estimated results of this trial are oriented at verifying whether a simplified TEE exam sequence can improve the efficiency of etiologies discrimination during CPB separation in cardiac surgery.

**Trial registration:**

ClinicalTrials.gov NCT05960552. Registered on 6 July 2023.

**Supplementary Information:**

The online version contains supplementary material available at 10.1186/s13063-024-08338-9.

## Administrative information

**Table:** 

Title {1}	Application of a Simplified Transesophageal Echocardiography Examination Sequence in High-risk Cardiac Surgery
Trial registration {2a and 2b}	ClinicalTrials.gov: NCT05960552
Protocol version {3}	24th August 2023-Final Version 2
Funding {4}	National High Level Hospital Clinical Research Funding (2022-PUMCH-B-007); the Chinese Academy of Medical Sciences (CAMS) Innovation Fund for Medical Sciences (2021-I2M-C&T-B-020)
Author details {5a}	^1^Department of Anesthesiology, Peking Union Medical College Hospital, Chinese Academy of Medical Sciences, Beijing, China ^2^Department of Cardiac Surgery, Peking Union Medical College Hospital, Chinese Academy of Medical Sciences, Beijing, China
Name and contact information for the trial sponsor {5b}	Chunhua Yu, E-mail: yuchh@pumch.cn
Role of sponsor {5c}	This is an investigator initiated clinical trial. The sponsor has approved the study design, and is response for collection, management, analysis, and interpretation of data; writing of the report; and the decision to submit the report for the publication

### Introduction

#### *Background and rationale* {6a}

Patients undergoing cardiac surgical procedures usually encounter a complexity of cardiopulmonary bypass (CPB) separation due primarily to hemodynamic compromise [1, 2]. The explainable determinants linked with difficult separation are multiple; increased age, critical comorbidities, redo cardiac surgery, poor cardiac function, a history of myocardial infarction, and complicated cardiac etiologies can all enhance the risk of its unexpectable onset [1]. When a difficult CPB separation is anticipated, not only more assisted inotropes and mechanical cardiac support are required [3–5] but also the clinical adverse consequences have been evidenced to be somewhat detrimental, such as high perils of morbidity and mortality [1]. Notably, there exists no standard protocol or guideline with regard to management of difficult CPB wean-off yet. The cause discrimination and rapid decision-making adoptions should therefore be equally emphasized in separation process.

Transesophageal echocardiography (TEE) utilized in the cardiac surgical scenario has been recognized as a predominant and reliable tool in revealing potential causes of failure to wean from CPB [2]. It can timely and efficiently provide vital clues, combined with compromised hemodynamic parameters, in identifying underlying reasons [2, 6, 7]. The vital role of intraoperative TEE has been fiercely unfolded out in surgical decision-making [8], peri-operative management [2], and clinical outcome improvements [9, 10].

Nevertheless, the conventional TEE scan sequence is time-consuming due to examinations without any restrictions of views, especially the rescue time the cardiac anesthesiologists and surgeons confronted with is relatively pushed. We initiate the program of perioperative rescue transesophageal echocardiography (PReTEE), attempting to simplify scan sequence and to focus collectively on a combination of three valuable views of midesophageal four chamber (ME 4C), midesophageal left ventricular long axis (ME LV LAX), and transgastric short axis (TG SAX). This simplified three-view TEE sequence derived objectively from clinical experience, in comparison with traditional TEE via a comprehensive or liberal examination, aims at detecting out the cardiac etiologies in a more prompt and accurate fashion.

### *Objectives* {7}

The primary objective is to assess whether PReTEE protocol can facilitate the TEE operators swiftly undertake an evaluation, as well as accurately discriminate the leading causes and accompanied cardiac pathologies in the phase of CPB separation, thereby yielding significant improvement regarding diagnostic efficiency.

### *Trial design* {8}

This is a single-center and randomized controlled trial which will be prospectively conducted at Peking Union Medical College Hospital, Beijing, China. The six TEE operators participating in this trial will be randomly assigned in a 1:1 fashion into the PReTEE group and the conventional TEE group. This protocol strictly conforms to the Standard Protocol Items Recommendation for Interventional Trials (SPIRIT) Checklist (Table 1).

**Table 1:** SPIRIT flow diagram. *PReTEE* perioperative rescue transesophageal echocardiography, *TEE* transesophageal echocardiography

	**Enrolment**	**Allocation**	**Post-allocation**	
Timepoint**	**Pre-allocation** **-t**_**1**_	**Preoperative** **0**	**Procedure** **t**_**1**_	***t***_***2***_
Enrolment:				
Eligibility screen	X			
Informed consent	X			
Allocation		X		
Interventions:				
PReTEE			X	X
Conventional TEE			X	X
Assessments:				
Outcome measurements			X	X
Adverse event evaluation			X	X

## Methods: participants, interventions and outcomes

### *Study setting* {9}

Our trial will be implemented from August 1, 2023, to August 1, 2025, at the Peking Union Medical College Hospital, a tertiary hospital in Beijing, China. Patient selection will be initiated following reviewing electronic system the day before surgery, based primarily on the inclusion and exclusion criteria. Then in cardiac wards, a detailed introduction of this trial as well as salutary and harmful effects should be reached out to the candidate patients in a popular and understood way. The informed consents must be signed if patients decide to participate in this trial owning to their willingness. The investigators in charge of patient recruitment should solve puzzles regarding this trial raised by patients.

### *Eligibility criteria* {10}

A patient who fulfills all of the following criteria will be included:

1. Male or female patients aged ≥ 18 years.
2. Patients who will undergo elective cardiac surgery with CPB.
3. Patients with high-risk profile as one of the followings: left ventricular ejection fraction of 50% and fewer, coronary artery bypass graft concomitant with valve procedures, multiple valve procedures, Euroscore > 6 or re-do cardiac surgery.
4. Patients who sign the informed consent to participate in the study.

The exclusion criteria are listed as follows, and a patient who meets any of the criteria will not be considered.

1. Gastrointestinal surgery history.
2. Active upper gastrointestinal bleeding.
3. Esophageal abnormality: stricture, tumor, fistula/ulcer, diverticulum, or recent perforation.
4. Non-elective cardiac procedures.
5. The requirement for mechanical cardiac support (extracorporeal membrane oxygenation, intra-aortic balloon pump, left ventricular assisted device) prior to cardiac surgery.

### *Who will take informed consent* {26a}

Patients who fulfill the inclusion and exclusion criteria after assessment will be invited to participate in this clinical trial in cardiac wards the day before cardiac surgery. The chief investigator will present detailed descriptions of purpose, trial protocol, and likelihood harms and benefits. Any query raised by eligible candidates must be solved as well. Informed consent, given in writing, must be given owning to patient willingness, and any withdrawal from this study at any time at patient’s own request must be allowed. A unique number will be assigned to patients who are ultimately enrolled into our program. Additionally, TEE operators participating in this trial are required to sign the informed consent.

### Additional consent provisions for collection and use of participant data and biological specimens {26b}

N/A, because no biological specimens or data associated will be collected in this trial.

## Interventions

### Explanation for the choice of comparators {6b}

Six TEE operators, willing to participate in this trial, are all junior cardiac anesthesiologist fellows at our institute whose accumulation of TEE performances are relatively comparable. They will be randomly assigned into the TEE groups stratified by allocation prior to trial initiation, with three in each group. The three operators in the PReTEE group will be required to accomplish the TEE examinations using a simple sequence of ME 4C, ME AV LAX, and TG SAX. On the other hand, another three in the conventional cohort will perform TEE examinations in a liberal fashion, that is, without any restriction on scan sequence.

### Intervention description {11a}

General cardiac anesthesia induction and maintenance will be prescribed at the chief anesthesiologist’s discretion. CPB procedure at our institute complies with a standardized pattern. The separation of CPB is ended with the administration of protamine and the removal of the venous and arterial cannulas. All patients during intraoperative periods will receive a round of TEE examination before and after CPB routinely. Hemodynamic parameters and TEE findings are two major determinants in the process of CPB separation. Following the completion of surgical procedures, patients will be admitted to the intensive care unit for further advanced monitoring and therapy. The timing of transfer from intensive care unit to the general ward will depend on patient physical recovery status.

When the process of CPB separation initiated, the TEE probe is immediately inserted by the TEE operators. Then they are required to remove and rotate the probe and to promptly detect the leading causes of complex CPB separation. After the probe is well inserted, the time taken in seconds till TEE scan completion will be measured by the study recorder. The etiology interpretations and their corresponding TEE views will also be reported to the recorder by operators and simultaneously kept in a form of questionnaire (Supplemental file 1). The maximum time for TEE sequence scanning permitted is 120 s. The number of both junior TEE operators and eligible patients available at our institute are to some extent of limitation; therefore, TEE examination for each patient is not conducted by equal numbers of operators stratified by allocation groups. During patient examinations by operators and the expert, the TEE probe is kept still.

The successful diagnosis is defined as the correct cause of discrimination within the specified 120 s. If an operator does not report any diagnoses or reports the uncorrected ones, a failure will be assigned. All TEE examinations in both groups will be supervised by the expert, who guarantees the safety of this study but does not facilitate the image obtainment or interpretation. The TEE results within this trial will also be finalized by the expert. TEE operators who finish the sequence scan and diagnosis report will immediately leave the operating room, therefore precluded from observing the expert’s TEE examination. The TEE expert finally will rate the cause discrimination per examination by the operators with a 5-point scale system (1 point, absolutely wrong; 2 points, wrong; 3 points, average; 4 points, correct; and 5 points, absolutely correct) (Supplemental file 1). There is no application of scoring system on the clarity of TEE view acquisition, as the TEE operators participating in this trial have obtained TEE manual skills. During the study period, the TEE machine we employ is Philips Ultrasound iE33 and the probe is Philips Ultrasound X7-2t.

Following the completion of TEE operators’ assessment, the TEE expert will routinely perform a standard comprehensive TEE examination. The cause provided by the TEE expert will be directly communicated to the attending anesthesiologist and cardiac surgeons, independent from this study, referred as therapeutic approach.

### Criteria for discontinuing or modifying allocated interventions {11b}

During each TEE scan, the examination will be immediately halted if any adverse events resulted from TEE occur or have the potential to occur. If a sign of a rapid rescue emerges, the trial will also be halted, and the expert and anesthesiologists in charge will then take over instead.

### Strategies to improve adherence to interventions {11c}

The TEE operators and the expert will be comprehensively trained before the initiation of this trial. Further, chief anesthesiologists will be informed of the trial protocol aimed at being not allowed to interpret the trial.

### Relevant concomitant care permitted or prohibited during the trial {11d}

All patients included will receive standard anesthetic plan in the operating room and post-operative treatment in the intensive care unit and general ward.

### Provisions for post-trial care {30}

Eligible patients will receive at least two rounds of intraoperative TEE scans and treatment for TEE-associated complications for free.

### Outcomes {12}

#### Primary endpoint

The primary endpoint we define is the difference in terms of correctly discriminating causes in the phase of CPB separation within specified 120 s between the PReTEE group and the conventional TEE group. A correct diagnosis by TEE is defined as the extent of agreement up to 70% and greater between the conventional TEE/PReTEE and the expert TEE; otherwise, an uncorrected diagnosis would be accounted for.

#### Secondary endpoints

This trial has eight secondary endpoints to be further assessed, which are listed as follows:

1. Detection rate of hypovolemia
  Hypovolemia is defined as a small left ventricular cavity size associated with normal or hyperdynamic global left ventricular systolic function.
2. Detection rate of outflow tract obstruction
  The obstruction we will observe includes pulmonary embolism or left ventricular outflow tract obstruction. For pulmonary embolism, thrombus mass visualized in the pulmonary artery, right atrium/ventricular is a direct indication but relatively rare; Indirect sign from TEE finding may be concluded as a dilated right atrium/ventricle with right ventricular akinesia. Left ventricular outflow tract obstruction is the occurrence of systolic anterior motion of the mitral valve accompanied by mitral regurgitation or not.
3. Detection rate of regional wall motion abnormality
4. Detection rate of left ventricular systolic dysfunction
  Dysfunction will be assessed via direct eyeballing with an indication of reduced or absent left ventricular systolic wall motion and systolic wall thickening. Grades of dysfunction (mild, moderate, and severe) will also be subsequently estimated.
5. Detection rate of right ventricular systolic dysfunction
  Right ventricular enlargement, severely decreased or absent right ventricular free wall endocardial excursion or wall thickening, or a D-shaped left ventricular cavity were all indications of right ventricular dysfunction.
6. Detection of decreased peripheral vascular resistance
7. Detection of valvular abnormalities
  Detailed information of valvulopathy in terms of valve categories and corresponding lesions will be additionally recorded.
8. The incidence of TEE-associated complications
  Major complications caused by intraoperative TEE probe insertion, manipulation and rotation include gastrointestinal bleeding or tearing or perforation. Moreover, the minor complications we will identify include lip or dental injuries, oral trauma/bleeding, odynophagia, and swallowing dysfunction.

## Safety evaluation

Safety evaluation accounted for in the study is composed of any TEE-linked adverse consequences, like esophageal perforations, pharyngeal trauma, and gastrointestinal bleed.

### Participant timeline {13}

Table 2 depicts the timeline designed for our trial.

**Table 2:** Participant timeline

Participate timeline
Electronic medical system
Eligibility screening
General cardiac ward
Written informed consent
A collection of baseline characteristics
Surgery
Intervention: The PReTEE group, the conventional group
The diagnosis efficiency regarding leading causes of difficult CPB separation
TEE-associated adverse complications Surgical data

### Sample size {14}

In our preliminary experiment, the time of finding out the cardiac etiologies in the PReTEE group was 36 s, whereas in the conventional group was 100 s. The preliminary results have never been published. Twenty PReTEE exams were performed using the available simulator employed at our center. Considering the apparent reduction of timing of diagnosis, no further PReTEE exams are planned to be conducted in cardiac surgical patients. However, one TEE scanning procedure in the conventional group was implemented for one cardiac patient in the operating room. To achieve a 90% power at a significance level of 5%, 42 TEE examinations should be conducted among eligible patients using a hazard ratio of 2.78. Considering a 20% dropout rate, a total of 46 TEEs are schemed to be consecutively performed. The sample size calculation was achieved using the gsDesign package via R software.

### Recruitment {15}

The recruitment process will take place the day before the surgery in the cardiac general ward, Peking Union Medical College Hospital, Beijing, China. Following manually screening the electronic health records, patient enrollment will be initiated according to the inclusion and exclusion criteria listed above.

## Assignment of interventions: allocation

### Sequence generation {16a}

The randomization of six TEE operators who are willing to participate in our trial will be accomplished via SASS software. They were evenly distributed in a 1: 1 ratio into the PReTEE group and conventional TEE group.

### Concealment mechanism {16b}

The concealment will be targeted for all TEE operators and investigators. The statistical analysts will also not be informed of the allocation groups until the final analysis is achieved.

### Implementation {16c}

The computerized randomization of TEE operators will be completed once the trial is started. Patient enrollment will be accomplished by the investigators.

## Assignment of interventions: blinding

### Who will be blinded {17a}

Enrolled patients, experts, data recorders, and outcome/data analysts are not aware of the TEE allocation groups. Further, the attending anesthesiologists and cardiac surgeons will also be blinded in the entire process of study conduction.

### Procedure for unblinding if needed {17b}

Unblinding is of necessity if applicable. The unblinded procedures will arise when threatening adverse events or esophageal injuries occur or this trial is completed.

### Data collection and management

 TEE operators are required to remove and rotate the probe and to promptly detect the leading causes of complex CPB separation. After the probe is well inserted, the time taken in seconds till TEE scan completion will be measured by the study recorder. The etiology interpretations and their corresponding TEE views will also be reported to the recorder and simultaneously kept in a form of questionnaire (Supplemental file 1). The maximum time for TEE sequence scanning permitted is 120 s. 

### Plans for assessment and collection of outcomes {18a}

Throughout the trial implementation, the primary and secondary outcomes of interest will be recorded in the form of questionnaires held by the trained recorder.

### Plans to promote participant retention and complete follow-up {18b}

No follow-up visits after patient hospital discharge are designed.

### Data management {19}

Signed informed consent and case report form (CRF) will be stored in a locked cabinet in our department. Patient baseline characteristics and surgical and postoperative information are all manually extracted from electronic database by trained recorders. Data mentioned above and TEE recordings are required to be entered twice into Excel. The rights of data access and process are only provided to authorized researchers, ethnic committees, and medical regulators. Any change to raw data must be noted with actual date and implementors. All statistical analyses should be completely retained in R software.

### Confidentiality {27}

The participants will be allocated an individual trial identification number. Aimed to protect the privacy of participants and patients, their information will be stored within a secure database. All data collected in the process of trial implementation should be stored by the investigators for 5 years. The rights of data access and process will only be accessible to study personnels, ethics committees, and medical regulators.

### Plans for collection, laboratory evaluation, and storage of biological specimens for genetic or molecular analysis in this trial/future use {33}

Not applicable. No biological specimens will be collected.

## Statistical methods

### Statistical methods for primary and secondary outcomes {20a}

The R software will be ultimately employed to perform all statistical analysis. The continuous data will be summarized as median (standard deviation) or median (interquartile range), whereas categorical data as number (percentage). Based on the time to event in the process of estimating the difference regarding diagnostic efficiency between the conventional TEE group and the PRe TEE group, a Cox proportional hazards model will be considered. In the Cox model, we will treat the TEE operators who failed the diagnosis as censored. A *P* < 0.05 will be considered statistically significant.

### Interim analyses {21b}

No plan for interim analyses will be considered in this trial, as the sample size we calculated is limited and patient risk is relatively low.

### Methods for additional analyses (e.g., subgroup analyses) {20b}

Subgroup analyses are not appropriate due to insufficient numbers of TEE scans.

### Methods in analysis to handle protocol non-adherence and any statistical methods to handle missing data {20c}

The loss regarding the times of TEE scan by operators in each cardiac surgical procedure will be controlled by adhering to the trial methods depicted above. We suppose that the number of missing data would be relatively limited. The dropout rate of 20% is also accounted for when calculating the sample size.

### Plans to give access to the full protocol, participant level-data, and statistical code {31c}

Upon reasonable request, the trial protocol, participant level-data, and statistical code can be available from the corresponding author via e-mails under hospital agreement.

## Oversight and monitoring

### Composition of the coordinating center and trial steering committee {5d}

This clinical trial will be performed by the Department of Anesthesiology at Peking Union Medical College Hospital. No coordinating center is required as this is a single-center trial. Additionally, there will be no stakeholder and public involvement group. The trial steering committee consisted of the ethics committee of the Peking Union Medical College Hospital, data monitoring committee, and the clinical research team.

The research team incorporates two principal investigators, a study coordinator, a data recorder, and a statistician. The team members meet twice a week to report the trial progress, identify issues, and then offer possible solutions.

### Composition of the data monitoring committee, its role, and reporting structure {21a}

Data monitoring is required by our Ethics Committee. The contents of data monitoring in this study will include medical records (baseline characteristics, surgical data, and postoperative outcome) and TEE-associated information. All data and records mentioned above will be kept in CRF. Missing data and outliers in the process of data collection, patient safety, and trial progress and integrity will all be assessed.

### Adverse event reporting and harms {22}

Mild esophageal injuries caused by TEE insertion and rotation will be documented and recorded in the CRF form. For severe adverse events, in addition to life rescue, the trial should be immediately haltered. All severe adverse events will be reported to Ethics Committee at our institute within 24 h of their development.

### Frequency and plans for auditing trial conduct {23}

The project management group will meet once a week to review the current status, assess the quality, and address existing problems. The trial steering group, including qualified experts of different medical disciplines, will meet annually to evaluate the implementation of the trial. Signed informed consent, case report form, incorrect data, and missing data will also be randomly examined by the steering group. To guarantee the quality, accuracy, and completeness of our data, data monitoring committee are responsible for data check.

### Plans for communicating important protocol amendments to relevant parties (e.g., trial participants, ethical committees) {25}

Any significant amendments with regard to study design, inclusion and exclusion criteria, sample size, interventions, and outcome assessments are regulated to be submitted to the website of Ethics Committee of Peking Union Medical College Hospital. Further, specific explanations and justifications for amendments must be presented in the process of authorization and scrutiny. Upon approval by the ethics committee, relevant amendments will also be explained in advance to the sponsor and funder as well as to the TEE operators, the expert, data recorders, and eligible patients who are involved in this trial. Any deviations from the protocol will be fully documented using a breach report form and proper state of noncompliance will be made. In addition, we will also update the protocol in the clinical trial registry.

### Dissemination plans {31a}

The ultimate results of our trial will be disseminated via peer-reviewed publications, academic conferences, teaching program, or other means.

## Discussion

We initiate this prospective trial to estimate the diagnostic proficiency of a simplified three-view TEE sequence in discriminating the leading causes and accompanied cardiac etiologies of a complex of CPB separation, especially in the clinical scenarios of unexplainable hemodynamic compromise. To the best of our knowledge, there has been no relevant publication on randomized controlled trials or with prospective nature, centered specifically on cardiac surgery, aimed at assessing the possible effects of a simplified TEE scan sequence.

A previous large-scale study has fiercely corroborated that patients characterized by high-risk profile benefited mostly from TEE exam in terms of morbidity and mortality reduction [9]. Accordingly, in this trial, we decided to enroll high-risk population, as they also frequently encounter a greater hazard of difficult CPB separation. When patients were confronted with critical sort of illness, this simplified TEE sequence is targeted to shrink diagnosing window without significant reduction on accuracy of pathologic identification. Our PReTEE protocol will also be preferred owning to its convenience to obtain and easiness to interpretate in practical scenarios. Furthermore, cardiac function and structural lesions are inclined to be mostly implicated by this simplified three-view sequence that we have created.

The sequence of TEE views prespecified in routine clinical work is never specific enough, which relies primarily on examiners’ accumulation of clinical judgment and experience. For novice residents and junior cardiac fellows, however, TEE scan performed in a liberal style may produce a comparable detection rate if sufficient time is permitted, but not in a rapid and swift fashion. The simple sequence we created can be embedded in the training programs centered on novice residents who are unfamiliar to TEE performance. Via this abbreviated TEE protocol, they can easily obtain skillful manipulation or proficient etiology discrimination ability, or both, especially when tricky cardiac issues emerge.

Another rationale during implementation of this trial should be emphasized. The diagnosis of cardiac pathologies in the phase of CPB separation will be finalized by the expert and subsequently provided to cardiac anesthesiologists in charge as the reference of targeted therapies; therefore, the clinical safety in the whole process of this trial can be guaranteed.

The major limitation of this study is the small number of TEE operators who will participate in this trial. The TEE operators are all junior cardiac anesthesiologists during the process of trial implementation, without any senior ones involved; it is not sure whether the clinical effects of PReTEE would fluctuate along with the experience of TEE operators. In addition, this is not a multi-center study and extrapolation of results will be limited.

## Trial status

Patient recruitment has started on 1 August 2023. The current version of this protocol is 2.0, released on 24 August 2023. We suppose the recruitment would be completed on 1 August 2025.

### Supplementary Information

Supplementary Material 1.

## Data Availability

Any data required to support the protocol can be supplied on request.

## Abbreviations

CAMS: Chinese Academy of Medical Sciences
CPB: Cardiopulmonary bypass
CRF: Case report form
ME 4C: Midesophageal 4 chamber
ME LV LAX: Midesophageal left ventricular long axis
PReTEE: Perioperative rescue transesophageal echocardiography
SPIRIT: Standard Protocol Items Recommendation for Interventional Trials
TEE: Transesophageal echocardiography
TG SAX: Transgastric short axis
