# Supplementary material for: Application of a simplified transesophageal echocardiography examination sequence in high-risk cardiac surgery
Source: Trials. 2024 Aug 13;25:535. doi: 10.1186/s13063-024-08338-9 (PMC11321141; doi:10.1186/s13063-024-08338-9)
Supplement: Supplementary file 1 — Supplementary Material 1. [file 13063_2024_8338_MOESM1_ESM.docx]

Supplemental file 1: The questionnaire which must be filled by the recorder in the operating room following participating TEE operators and expert complete the TEE examinations.

| TEE operators |  |  |
| --- | --- | --- |
| Time | _______seconds  Time taken in seconds from TEE probe movement and rotation to views acquisition |  |
| Diagnosis |  | TEE views |
| Hypovolemia | □Absent □Present |  |
| Decreased peripheral vascular resistance | □Absent □Present |  |
| Regional wall motion abnormality | □Absent □Present |  |
| Left ventricular systolic dysfunction (qualitatively) | □Absent □Present (□Mild □Moderate □Severe) |  |
| Right ventricular systolic dysfunction (qualitatively) | □Absent □Present (□Mild □Moderate □Severe) |  |
| Left ventricular outflow tract obstruction | □Absent □Present |  |
| Right ventricular outflow tract obstruction | □Absent □Present |  |
| Valvulopathy | □Absent □Present |  |
| Other findings |  |  |
| TEE expert |  |  |
| Diagnosis |  | TEE views |
| Hypovolemia | □Absent □Present |  |
| Decreased peripheral vascular resistance | □Absent □Present |  |
| Regional wall motion abnormality | □Absent □Present |  |
| Left ventricular systolic dysfunction (qualitatively) | □Absent □Present (□Mild □Moderate □Severe) |  |
| Right ventricular systolic dysfunction (qualitatively) | □Absent □Present (□Mild □Moderate □Severe) |  |
| Left ventricular outflow tract obstruction | □Absent □Present |  |
| Right ventricular outflow tract obstruction | □Absent □Present |  |
| Valvulopathy | □Absent □Present |  |
| Other findings |  |  |
| Five-point scale system by expert towards identification of TEE operators | □ 1-point, absolutely wrong  □ 2-point, wrong  □ 3-point, average  □ 4-point, correct  □ 5-point, absolutely correct |  |
